# Supplementary material for: Can the Cans: Determinants of Container Deposit Behavior before and after Introduction of a Container Refund Scheme
Source: Behav Sci (Basel). 2024 Feb 2;14(2):112. doi: 10.3390/bs14020112 (PMC10885914; doi:10.3390/bs14020112)
Supplement: Supplementary file 1 [file behavsci-14-00112-s001.zip › behavsci-2656898-supplementary.pdf]

Supplementary Table S1. *Pairwise Comparisons for each construct.*

Measure	Time Point Comparison	Mean Difference	SE	*p*
Attitude	1 -2	.047	.114	.679
	1 -3	.106	.085	.217
	2- 3	.058	.074	.431
Norm	1 -2	.175	.121	.151
	1 -3	.150	.134	.267
	2- 3	-.025	.100	.804
PBC	1 -2	.178	.107	.100
	1 -3	.356*	.108	.001
	2- 3	.178*	.074	.019
Intention	1 -2	.289*	.104	.007
	1 -3	.336*	.118	.005
	2- 3	.047	.070	.499
Behaviour	1 -2	.228	.128	.079
	1 -3	.556*	.127	<.001
	2- 3	.328*	.122	.009
Habit	1 -2	.250	.147	.093
	1 -3	.414*	.153	.008
	2- 3	.164	.113	.152
